# Japanese 2011 nationwide survey on complications from spine surgery

**DOI:** 10.1007/s00776-014-0656-6

**Published:** 2014-12-05

**Authors:** Yasuaki Imajo, Toshihiko Taguchi, Kazunori Yone, Atsushi Okawa, Koji Otani, Tadanori Ogata, Hiroshi Ozawa, Yoichi Shimada, Masashi Neo, Tetsuhiro Iguchi

**Affiliations:** 1 Department of Orthopaedic Surgery, Yamaguchi University Graduate School of Medicine, 1-1 Minami-kogushi, Ube, Japan; 2 Department of Physical Therapy, School of Health Sciences, Faculty of Medicine, Kagoshima University, Kagoshima, Japan; 3 Section of Orthopaedic and Spinal Surgery, Graduate School of Medical and Dental Science, Tokyo Medical and Dental University, Tokyo, Japan; 4 Department of Orthopaedic Surgery, Faculty of Medicine, Fukushima Medical University, School of Medicine, Fukushima, Japan; 5 Spine Center, Ehime University Hospital, Matsuyama, Japan; 6 Department of Orthopaedic Surgery, Tohoku University School of Medicine, Sendai, Japan; 7 Deptartment of Orthopedic Surgery, Akita University Graduate School of Medicine, Akita, Japan; 8 Department of Orthopedic Surgery, Osaka Medical College, Takatsuki, Japan; 9 Department of Orthopaedic Surgery, Hyogo Rehabilitation Center Hospital, Kobe, Japan

## Abstract

**Background:**

The Japanese Society for Spine Surgery and Related Research (JSSR) previously carried out two nationwide surveys in 1994 and 2001 on complications from spine and spinal cord surgery. More than 10 years have now elapsed since 2001. Rapidly ageing populations have major impacts on society, particularly in the medical field. The purpose of this study was therefore to examine the present situation for spine surgery in Japan.

**Methods:**

The JSSR research team prepared a computerized questionnaire made up of two categories in order to capture clinicopathological information and surgical information. A recordable optical disc for data storage was sent to surgeons who were certified for spine surgery by JSSR. The data was analyzed.

**Results:**

The JSSR carried out a nationwide survey of complications of 31,380 patients. Patients aged 60 years or older comprised 63.1 % of the overall cohort. This was considerably higher than observed in previous surveys. Degenerative spinal diseases increased 79.7 %. With regard to surgical approach, the incidence of anterior surgery has decreased, while that of posterior surgery has increased compared to the earlier surveys (both *p* < 0.05). Spinal instrumentation was applied in 30.2 % cases, compared to 27.0 and 34.0 % cases in the 1994 and 2001 surveys, respectively. Intraoperative and postoperative complications were reported in 10.4 % and were slightly increased compared to 8.6 % in the earlier surveys (both *p* < 0.05). Diseases associated with a high incidence of complication included intramedullary tumor (29.3 %) and primary malignant tumor (22.0 %). The highest incidence of complication was dural tear (2.1 %), followed by neurological complication (1.4 %).

## Introduction

The practice of spine surgery has undergone rapid changes in Japan. The ageing population has led to an increase in the number of high-risk patients for surgery, including those with comorbidities and/or compromised immunity. Consequently, the use of less invasive practices such as endoscopic and microscopic surgery has increased [1]. At the end of 2010, 23.1 % of the Japanese population was aged > 65 years and 11.4 % was aged > 75 years. By 2025, it is estimated that 30.5 % of the population will be aged > 65 years [2]. With the exception of Germany and Italy, the majority of European countries have less than 18 % of their population aged > 65 years [3]. Rapidly ageing populations have major impacts on society, particularly in the medical field.

The Japanese Society for Spine Surgery and Related Research (JSSR) forms part of the Japanese Orthopaedic Association (JOA) and comprises 3,611 spine surgeons and researchers. The JSSR previously carried out two nationwide surveys in 1994 and 2001 on complications from spine and spinal cord surgery [4, 5]. The results from these surveys were helpful to surgeons in preventing pitfalls in spine and spinal cord surgery. They also helped to inform the general public about spine surgery and assisted patients with understanding informed consent.

Although there have been some publications on spinal complications in large patient cohorts, the results are often limited to patients from a single hospital or from a small district [6]. Recently, the Scoliosis Research Society published results on spinal complications [7]. However, with the exception of our two previous surveys, to our knowledge, there is little literature that reports on nationwide surveys of spinal complications [8]. More than 10 years have now elapsed since the last Japanese nationwide survey conducted in 2001. The purpose of this study was therefore to examine the present situation for spine surgery in Japan, including factors such as patient characteristics, surgical approaches, instruments and materials used, and the frequency and nature of complications. Comparison with our previous survey results is likely to reveal important trends for spine surgery in a country with a rapidly ageing population.

## Materials and methods

### Data collection

This survey aimed to enroll all patients who underwent spine surgery in Japan during the one-year period from 1 January 2011 to 31 December 2011. The JSSR research team prepared a computerized questionnaire made up of two categories in order to capture clinicopathological information and surgical information. In January 2012, a recordable optical disc for data storage was sent to 1,105 surgeons who were certified for spine surgery by JSSR. This data was returned by the end of May 2012.

### Clinicopathological and surgical data

The clinicopathological variables that were investigated included patient information [age, gender, body weight, height, body mass index (BMI)], diabetes mellitus (DM), dialysis, corticosteroid use, disease-modifying anti-rheumatic drug (DMARD) therapy involving biological therapy, and Parkinson’s disease. Other requested information included the involved classification of spinal involvement, degeneration, deformity, ossified lesions, spondylolisthesis, inflammation, infection and tumors.

The requested surgical information included surgical approaches, intraoperative blood loss, operation time, surgical technology, decompression methods, fusion methods and instrumentation. The 22 reported items for intraoperative and postoperative complications in hospital are listed in Table 1. Information regarding the experience of the principal surgeon who operated on the patient was also collected. This was classified as 1–4 years of surgical experience after graduation, 5–9 years experience, 10–19 years experience, or 20 or more years of experience.

**Table 1:** Items of intraoperative and postoperative complications

Nerve root damage
Spinal cord damage
Cauda equina damage
Dural tear
Cerebrospinal fluid (CSF) leakage
Wrong level
Implant failure
Implant dislodgment
Vascular injury
Deep wound infection
Epidural hematoma
Pulmonary embolism/thromboembolism
Mental disorder
Hemothorax/pneumothorax
Circulatory disease
Cerebral disease
Digestive disease/liver disease
Anesthesiological
Respiratory disease
Urinary disease
Death
Others

The above items were evaluated and compared with data from previous surveys. Complications were evaluated in relation to the surgical approach, instrumentation surgery, surgeon and BMI. Associations between intraoperative blood loss and the incidence of deep wound infection (DWI), epidural hematoma (EH), death and pulmonary embolism and/or thromboembolism (PE/TE) and operation time were also examined.

### Statistical analysis

Statistical analyses were performed using Statcel 2. For categorical variables, cross-tabulations were made and a Chi square test was used for comparison of proportions. For continuous variables, statistical significance was assessed using the *F* test, Welch’s *t* test, Student’s *t* test for comparison of two means, and Spearman’s correlation coefficient for association between quantitative characteristics. A *p* value of <0.05 was considered significant in all the analyses. In order to evaluate factors associated with major intraoperative and postoperative complications, multiple logistic regression analyses were performed by use of a computer program: Statflex 6.0 (Artech Co., Ltd., Osaka, Japan. URL: http://www.statflex.net/).

This survey received approval from the institutional review board of Yamaguchi University Hospital.

## Results

### Basic information

The certified surgeons were distributed in 750 institutions nationwide and a response was achieved from 209 institutions (response rate 28 %). Of these, 63 (30 %) were university hospitals. Following the exclusion of cases that lacked clinicopathological or surgical information, data was available for a total of 31,380 patients, comprising 18,546 men, 12,747 women and 87 persons of unknown gender. This cohort was considerably larger than for both the 1994 (19,271 cases) and 2001 (16,157 cases) surveys [4, 5].

### Age

**Fig. 1:**
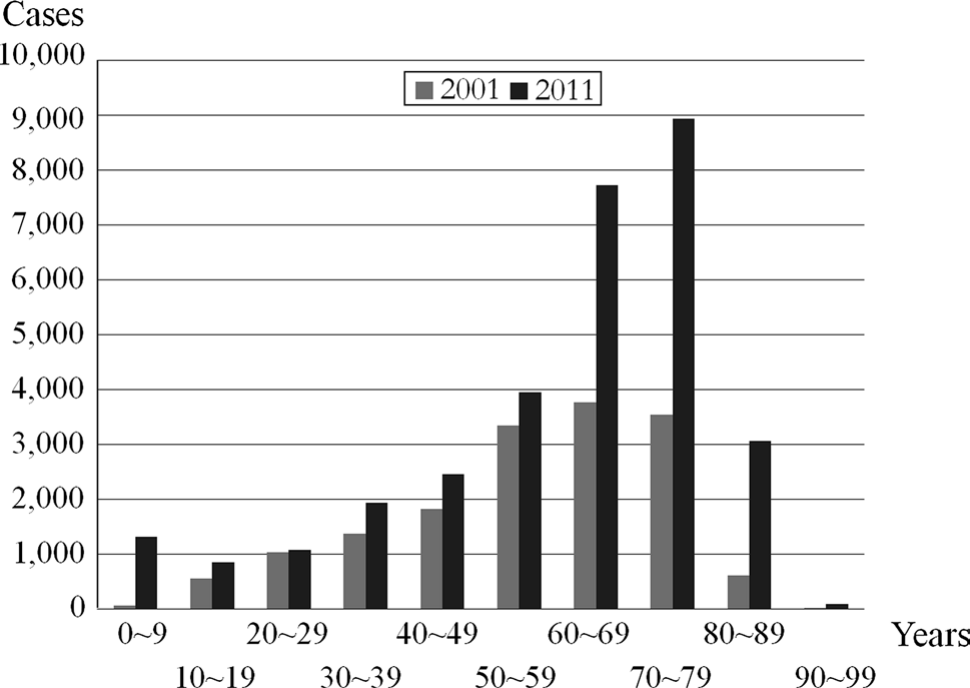
Distribution of cases according to patient age in the 2001 and 2011 surveys

The mean age was 59.3 years (range 0–97 years) and the most frequent age was in the 70–79 year range (Fig. 1). This is older than the most common age reported in the 1994 and 2001 surveys [4, 5]. A total of 19,802 patients were aged 60 years or older, corresponding to 63.1 % of the overall cohort. This was higher than in the 1994 and 2001 surveys (37.3 and 49.0 %, respectively), demonstrating a marked increase in the proportion of elderly patients who underwent spine surgery (*p* < 0.05 for 2001 vs. 2011) [4, 5]. In 2001, just 3.8 % (617) of patients were aged 80 years or older compared to 10.0 % (3,136 patients) in 2011 (*p* < 0.05) [5].

### Experience of the surgeons

In 2011, 0.8 % of operations were performed by surgeons with 1–4 years of experience after graduation, 8.8 % by surgeons with 5–9 years of experience, 42.1 % by surgeons with 10–19 years of experience and 48.2 % by surgeons with 20 or more years of experience. In all, 90.3 % (28,352) of patients were operated by surgeons with 10 or more years of experience, compared to 83.1 % (12,760) in 2001 (*p* < 0.05) [5].

### Preoperative complications

DM was reported in 3,792 patients (12.1 %), dialysis in 437 (1.4 %), corticosteroid use in 701 (2.2 %), DMARD therapy involving biological therapy in 512 (1.6 %) and Parkinson’s disease in 198 (0.6 %).

### Disease information

Table 2 shows the categories and subcategories of diagnosis. The category of “degenerative disease” was the most frequent diagnosis, corresponding to 79.7 % of all cases. Amongst the subcategories, stenosis was the most common (40.5 %), followed by disc herniation (23.0 %). This compares with the 1994 survey in which disc herniation was the most common (38.5 %), and the 2001 survey in which stenosis was the most common (31.1 %) [4, 5]. Other frequent diagnoses were tumor (5.4 %), trauma (2.9 %), osteoporotic vertebral collapse (2.3 %), inflammation (2.9 %) and spinal deformity (6.8 %). The contribution of degenerative disease (79.7 %) was higher than that reported in the 1994 and 2001 surveys (78.7 and 78.2 %, respectively) [4, 5]. The frequencies of osteoporotic vertebral collapse and stenosis were higher than those reported in the 2001 survey (*p* < 0.05), reflecting the higher proportion of elderly patients undergoing spine surgery [5]. Spinal deformity (6.7 %) was more frequent than that reported in the 1994 and 2001 surveys (2.1 and 2.3 %, respectively; *p* < 0.05), reflecting the increased use of spinal instrumentation [4, 5]. The frequencies of disc herniation, ossification of ligaments, metastatic spine tumor and RA were lower than those reported in the 2001 survey (each *p* < 0.05) [5].

**Table 2:** Diagnosis and incidence

Category	Subcategory	No. of cases incidence (%)		
		2011	2001	1994
Degenerative disease	Disc herniation	7,964 (23.0)	4,385 (27.1)	– (38.5)
	Spondylolysis, isthmic spondylolisthesis	556 (1.6)	371 (2.3)	– (3.7)
	Degenerative spondylolisthesis	3,214 (9.3)	1,423 (8.8)	– (6.4)
	Stenosis	14,001 (40.5)	5,021 (31.1)	– (24.5)
	Ossification of ligaments	1,434 (4.2)	857 (5.3)	– (5.6)
	Others	388 (1.1)	580 (3.6)	–
Tumor	Primary benign spinal tumor	674 (1.9)	147 (0.9)	– (0.8)
	Primary malignant spinal tumor	82 (0.2)		
	Metastatic spinal tumor	389 (1.1)	320 (2.0)	– (2.1)
	Intramedullary tumor	75 (0.2)	61 (0.4)	– (3.1)
	Intradural extramedullary tumor	471 (1.3)	323 (2.0)	
	Extradural tumor	240 (0.7)	117 (0.7)	
	Cauda equina tumor	–	83 (0.5)	
Trauma		1,014 (2.9)	905 (5.6)	– (4.8)
Osteoporotic vertebral collapse		813 (2.3)	186 (1.2)	–
Inflammation	Pyogenic infection	521 (1.5)	292 (1.8)	– (1.0)
	Rheumatoid arthritis	240 (0.7)	264 (1.6)	– (1.4)
	Tuberculous infection	55 (0.2)	69 (0.4)	– (0.5)
	Fungal infection	6 (0.0)	–	–
	Seronegative arthritis without AS	5 (0.0)	–	–
	Ankylosing spondylitis (AS)	11 (0.0)	–	–
	Dialysis	101 (0.3)	–	–
	Others	48 (0.2)	39 (2.4)	–
Spinal deformity	Scoliosis	1,485 (4.3)	327 (2.0)	– (1.8)
	Kyphosis	537 (1.6)	60 (0.3)	– (0.3)
	Combined	169 (0.5)	–	–
	Others	120 (0.4)	–	–
Metabolic bone disease		–	9 (0.1)	– (0.1)
General affection of bone		–	22 (0.1)	– (0.1)
Others		–	296 (1.9)	– (5.0)

### Degenerative disease 

Table 3 shows details of the cases with degenerative disease. A total of 4,954 patients underwent spine surgery for cervical disease: 3,564 for cervical spondylotic myelopathy or radiculopathy, 641 for cervical disc herniation and 149 for ossification of the posterior longitudinal ligament (OPLL). A total of 21,338 patients underwent spine surgery for lumbar disease: 11,136 for lumbar spinal stenosis, 7,086 for lumbar disc herniation, 1,999 for degenerative spondylolisthesis and 270 for spondylolysis or isthmic spondylolisthesis.

**Table 3:** Spine surgery for degenerative diseases

Category	Subcategory	No. of cases incidence (%)		
		2011	2001	1994
Cervical spine	Cervical spondylotic myelopathy/radiculopathy	3,564 (72.0)	1,800 (55.0)	1,625 (44.3)
	Cervical disc herniation	641 (12.9)	475 (14.5)	1,046 (28.6)
	OPLL	149 (3.0)	598 (18.3)	757 (20.7)
	Other degenerative diseases	600 (12.1)	396 (12.1)	235 (6.4)
Lumbar spine	Lumbar disc herniation	7,086 (33.2)	3,753 (43.0)	6,156 (55.2)
	Lumbar spinal stenosis	11,136 (52.2)	3,042 (34.9)	2,953 (26.5)
	Degenerative spondylolisthesis	1,999 (9.4)	1,382 (15.8)	1,169 (10.5)
	Spondylolysis, isthmic spondylolisthesis	270 (1.3)	359 (4.1)	687 (6.2)
	OPLL	15 (0.1)	20 (0.2)	47 (0.4)
	OLF	113 (0.5)		
	OPLL + OLF	8 (0.0)		
	Other degenerative diseases	711 (3.3)	169 (1.9)	138 (1.2)

*OPLL* ossification of posterior longitudinal ligament, *OLF* ossification of ligamentum flavum

### Surgical approaches

**Fig. 2:**
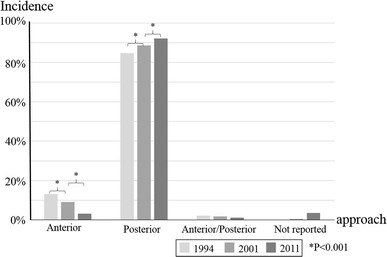
Incidence of surgical approach in the 1994, 2001 and 2011 surveys

Information on the surgical approach was available for 30,271 cases (96.5 % of total). Anterior surgery was indicated in 1,012 cases (3.2 %), posterior surgery in 28,909 cases (92.1 %) and combined anterior/posterior surgery in 350 cases (1.1 %). In the 1994 survey, anterior surgery was indicated in 13.2 %, posterior surgery in 84.7 % and combined anterior/posterior surgery in 2.2 %, while in the 2001 survey, anterior surgery was indicated in 9.1 %, posterior surgery in 88.6 % and combined anterior/posterior surgery in 1.8 % [4, 5]. Compared to the earlier surveys, the frequency of anterior surgery has decreased while the incidence of posterior surgery has increased (Fig. 2) [4, 5].

### Degenerative disease in cervical spine

Table 4 shows the main surgical approaches for degenerative disease. In the 2011 survey, anterior surgery for patients with cervical spondylotic myelopathy or radiculopathy (CSM or CSR) and cervical disc herniation (CDH) had decreased to 7.1 % (248) and 47.9 % (297), respectively (each *p* < 0.05), compared to the 2001 survey [5]. In the 2001 survey, anterior surgery was indicated in 12.8 % (76) of patients with cervical OPLL, whereas in the 2011 survey this had decreased to 11.6 % (17; *p* = NS) [5]. In the 2011 survey, posterior surgery for patients with CSM or CSR and CDH increased to 91.8 % (3,208) and 49.7 % (308), respectively (each *p* < 0.05), compared to the 2001 survey [5]. In the 2001 survey, posterior surgery was indicated in 85.6 % (510) of patients with cervical OPLL, whereas in the 2011 survey this increased to 88.4 % (130; *p* = NS) [5].

**Table 4:** Surgical approaches for degenerative diseases

Degenerative disease	No. of cases incidence (%)								
	Anterior			Posterior			Antero/posterior		
	2011	2001	1994	2011	2001	1994	2011	2001	1994
Cervical spondylotic myelopathy/radiculopathy	248 (7.1)	243 (13.6)	– (21)	3,208 (91.8)	1528 (85.3)	– (75)	39 (1.1)	21 (1.2)	– (4)
Cervical disc herniation	297 (47.9)	358 (75.8)	– (52)	308 (49.7)	113 (23.9)	– (44)	15 (2.4)	1 (0.2)	– (4)
Cervical OPLL	17 (11.6)	76 (12.8)	–	130 (88.4)	510 (85.6)	–	0 (0.0)	9 (1.5)	–
Lumbar disc herniation	8 (0.1)	36 (1.0)	– (4)	6,826 (99.8)	3579 (98.9)	– (95)	9 (0.1)	3 (0.1)	– (1)
Lumbar spinal stenosis	5 (0.0)	–		10,691 (99.7)	–	–	25 (0.2)	–	–
Lumbar degenerative spondylolisthesis	3 (0.2)	8 (0.6)	– (13)	1,949 (99.6)	1343 (98.7)	– (85)	4 (0.2)	9 (0.7)	– (2)
Lumbar spondylolysis, isthmic spondylolisthesis	0 (0.0)	4 (1.2)		259 (98.9)	335 (97.4)		3 (1.1)	5 (1.5)	
Lumbar OPLL	0 (0.0)	–		13 (100)	–		0 (0.0)	–	
Lumbar OLF	0 (0.0)	–		112 (100)	–		0 (0.0)	–	
Lumbar OPLL + OLF	0 (0.0)	–		8 (100)	–		0 (0.0)	–	

*OPLL* ossification of posterior longitudinal ligament, *OLF* ossification of ligamentum flavum

### Degenerative disease in lumbar spine

Posterior surgery was indicated for almost all patients with lumbar degenerative disease: 99.8 % with lumbar disc herniation, 99.7 % with lumbar spinal stenosis, 99.6 % with degenerative spondylolisthesis, 99.8 % with spondylolysis and isthmic spondylolisthesis, and 100 % with ossification of ligaments. Posterior surgery for all degenerative diseases was more frequent than in both earlier surveys [4, 5].

### Operation time

Information on the operation time was available for 31,235 (99.5 %) cases. The operation time was less than 1 h in 3,899 cases (12.5 %), 1–2 h in 10,806 cases (34.6 %), 2–3 h in 8,060 cases (25.8 %), 3–4 h in 4,115 cases (13.2 %), 4–5 h in 2,070 cases (6.6 %), 5–6 h in 1,030 cases (3.3 %), 6–8 h in 851 cases (2.7 %), 8–10 h in 300 cases (1.0 %) and more than 10 h in 104 cases (0.3 %). Thus, in 72.9 % of patients, the operation time was 3 h or less.

### Intraoperative blood loss

Information on intraoperative blood loss was available for 31,188 (99.4 %) cases. This was 200 ml or less in 21,752 cases (69.7 %), 200–500 ml in 5,445 cases (17.5 %), 500–1,000 ml in 2,079 cases (6.7 %), 1,000–2,000 ml in 839 cases (2.7 %), 2,000 ml or more in 391 cases (1.2 %) and an unknown volume in 682 cases (2.2 %).

### Surgical procedures

Information on surgical procedures was available for 4,665 (94.2 %) of the patients with cervical degenerative disease. Decompression was commonly indicated for cervical spondylotic myelopathy or radiculopathy (81.0 %) and for cervical OPLL (80.3 %). Decompression and fusion were indicated for cervical disc herniation (50.2 %). Information was available for 21,011 (98.5 %) cases with lumbar degenerative disease. Decompression was mostly indicated for lumbar spinal stenosis (68.7 %) and for lumbar disc herniation (93.2 %), while decompression and fusion were indicated for lumbar degenerative spondylolisthesis (68.4 %).

### Surgical technology

Table 5 shows the frequencies of use for the different surgical technologies. The number cases treated by endoscopic surgery in the 2011 survey was more than tenfold higher than in the 2001 survey, while the number of cases treated by microscopic surgery increased about fourfold [5]. In the 2001 survey, endoscopic and microscopic surgery were indicated in 2.6 and 11.1 % of cases, respectively, whereas in the 2011 survey these had increased to 12.9 and 20.5 %, respectively (*p* < 0.05) [5].

**Table 5:** Surgical technologies

Surgical technology	No. of cases frequency (%)	
	2011	2001
Conventional	20,697 (65.0)	–
Endoscope	4,110 (12.9)	387 (2.6)
Microscope	6,523 (20.5)	1,651 (11.1)
Percutaneus	501 (1.6)	–

### Spinal instrumentation

Spinal instrumentation was applied in 9,487 (30.2 %) of the 31,380 cases in the 2011 survey, compared to 5,210 (27.0 %) and 5,497 (34.0 %) cases in the 1994 and 2001 surveys, respectively [4, 5]. Thus, the actual number of cases with spinal instrumentation was about 4,000 more than in both previous surveys. A pedicle screw system was used in the majority (82.3 %) of patients (Table 6). This was higher than the frequency reported in the 1994 (63 %) and 2001 (54.6 %) surveys [4, 5]. Surgeons with less than 5 years experience performed spinal instrumentation in 16.0 % (40/251 cases), while surgeons with 5–9, 10–19 or more than 20 years experience performed spinal instrumentation at a similar frequency of 30.2 % (831/2,754 cases), 30.3 % (4,005/13,212 cases) and 30.4 % (4,606/15,140 cases), respectively. Table 7 shows the frequencies of instrumentation surgery used for different diseases. This procedure was commonly indicated for spinal deformity (74.1–81.1 %), lumbar spondylolysis and isthmic spondylolysis (77.4 %), and RA (75.4 %). Overall, these frequencies were similar to those reported in the two earlier surveys for the same conditions [4, 5].

**Table 6:** Use of implants

Implant	No. of cases frequency (%)		
	2011	2001	1994
Interbody fusion with cage (anterior)	335 (3.5)	1,452 (26.4)	–
Interbody fusion with cage (posterior)	3,686 (38.9)		–
Vertebral body replacement by cage	154 (1.6)		–
Plate	532 (5.6)	–	–
Cervical anterior plate		300 (5.5)	–
Thoracolumbar anterior plate		289 (5.3)	–
Rod + pedicle screw	7,496 (79.0)	3,004 (54.6)	3,264 (63)
Plate + pedicle screw	311 (3.3)		
Facet screw	8 (0.1)	–	
Transarticular screw	50 (0.5)	–	
Rod + lamina hook	455 (4.8)	418 (7.6)	744 (14)
Rod + pedicle hook	133 (1.4)		
Rod + hook	–		
Rod + hook, wire	–	–	
Rod + lateral mass screw	372 (3.9)	–	
Plate + lateral mass screw	12 (0.1)	–	
Odontoid screw	18 (0.2)	–	–
Wiring	–	253 (4.6)	501 (10)
Others	1,047 (11.0)	1,233 (22.4)	701 (13)

**Table 7:** Frequencies of instrumentation surgery

Disease	No. of cases frequency (%)		
	2011	2001	1994
Cervical disc herniation	197 (30.2)	450 (10.3)	– (10)
Lumbar disc herniation	470 (6.5)		
Thoracic OPLL	22 (48.9)	–	–
Thoracic OLF	51 (22.3)	–	–
Thoracic OPLL + OLF	13 (59.1)	–	–
Lumbar spondylolysis, isthmic spondylolisthesis	428 (77.4)	–	–
Lumbar degenerative spondylolisthesis	2,124 (67.9)	–	–
Lumbar spinal stenosis	3,594 (30.9)	–	–
Lumbar OPLL	19 (51.4)	–	–
Lumbar OLF	27 (17.1)	–	–
Lumbar OPLL + OLF	13 (59.1)	–	–
Tumor			
Primary benign	111 (16.5)	66 (44.9)	– (40)
Primary malignant	27 (32.9)		
Metastatic	254 (65.3)	236 (73.8)	– (81)
Intramedullary	1 (1.3)	–	–
Inflammation			
Pyogenic	99 (19.0)	68 (23.3)	– (20)
RA	181 (75.4)	224 (84.8)	– (85)
Tuberculous	25 (45.5)	33 (47.8)	– (37)
Fungal	1 (16.7)	–	–
Seronegative arthritis^a^	3 (60.0)	–	–
AS	8 (72.7)	–	–
Dialysis	53 (52.5)	–	–
Others	13 (27.1)	–	–
Spinal deformity			
Scoliosis	1,100 (74.1)	289 (88.4)	– (92)
Kyphosis	430 (80.1)	48 (80.0)	– (74)
Combined	137 (81.1)	–	–
Others	49 (40.8)	–	–

*OPLL* ossification of posterior longitudinal ligament, *OLF* ossification of ligamentum flavum, *RA* rheumatoid arthritis, *AS* ankylosing spondylitis

^a^Excluding AS

### Complications

Table 1 shows the intraoperative and postoperative items of complication. These were reported in 3,269 of the 31,380 cases (10.4 %) in the 2011 survey and were significantly higher than in the 1994 (8.6 %, 1,569/19,271) and 2001 (8.6 %, 1,383/16,157) surveys (both *p* < 0.05) [4, 5]. The mean patient age for patients with intraoperative and postoperative complications in the 2011 survey was 64.4 ± 17.0 years, while it was 58.9 ± 20.6 years for those without complications (*p* < 0.01).

### Intraoperative and postoperative complications according to diagnosis

Table 8 shows the incidence of intraoperative and postoperative complications according to diagnosis. Diseases accompanied by a high incidence of complications included intramedullary tumor, primary malignant tumor, osteoporotic vertebral collapse, inflammatory disease and spinal deformity.

**Table 8:** Intraoperative and postoperative complications

Disease	Incidence		
	Intraoperative and postoperative complications		
	2011	2001 (%)	1994 (%)
Disc herniation	5.6 % (447/7,964)	5.0	4.2
Lumbar spondylolysis and isthmic spondylolysis	13.1 % (73/556)	10.0	11.5
Lumbar degenerative spondylolisthesis	10.7 % (345/3,214)	8.9	12.2
Stenosis	10.8 % (1513/14,001)	7.7	6.6
Ossification of ligament	15.0 % (215/1,434)	13.4	11.0
Tumor			
Primary benign	15.3 % (103/674)	12.2–12.9	22.0
Primary malignant	22.0 % (18/82)		
Metastatic	18.3 % (71/389)		
Intramedullary	29.3 % (22/75)	6.0–18.0	12.3
Intradural extramedullary	16.1 % (76/471)		
Epidural	12.9 % (31/240)		
Osteoporotic vertebral collapse	20.9 % (170/813)	19.4	
Inflammation			
Pyogenic	13.8 % (72/521)	14.4–15.9	13.9
RA	17.9 % (43/240)		19.3
Tuberculous	18.2 % (10/55)		19.2
Fungal	0.0 % (0/6)		–
Seronegative arthritis^a^	60.0 % (3/5)		–
AS	45.5 % (5/11)		–
Dialysis	20.8 % (21/101)		–
Others	8.3 % (4/48)		–
Spinal deformity			
Scoliosis	16.5 % (245/1,485)	15.6–16.7	15.7
Kyphosis	25.3 % (136/537)		31.0
Combined	20.7 % (35/169)		–
Others	8.3 % (10/120)	–	

*OPLL* ossification of posterior longitudinal ligament, *OLF* ossification of ligamentum flavum, *RA* rheumatoid arthritis, *AS* ankylosing spondylitis

^a^Excluding AS

### Neurological and non-neurological complications in representative diseases

Table 9 shows the frequencies of neurological and non-neurological complications in representative diseases. Neurological complications comprised spinal cord damage, nerve root damage and cauda equine damage. The incidence of neurological complications was very low for cases with disc herniation (0.6 %), stenosis (1.1 %), spondylolisthesis (1.1 %) and osteoporotic vertebral collapse (1.0 %), but was higher for cases with ossification of ligaments (3.7 %) and spinal deformity (2.1–2.8 %).

**Table 9:** Neurological and non-neurological complications in representative diseases

	Incidence					
	2011		2001		1994	
	NC (%)	NNC (%)	NC (%)	NNC (%)	NC (%)	NNC (%)
Disc herniation	0.6	4.3	1.0	4.0	0.8	2.5
Stenosis	1.1	8.1	1.8	5.9	1.1	4.2
Spondylolisthesis	1.1	8.4	0.9	8.0	1.7	7.3
Ossification of ligaments	3.7	9.3	3.6	9.8	2.4	5.6
Spinal deformity						
Scoliosis	2.1	12.5	0.3	16.4	1.7	11.4
Kyphosis	2.8	19.2			3.3	21.7
Osteoporotic vertebral collapse	1.0	15.9	1.1	18.3	–	–

*NC* neurological complications, *NNC* non-neurological complications

### Relationship between the incidence of complication and the level of spine

Complications occurred in 12.0 % of cases treated at the cervical level, in 14.8 % of those treated at the thoracic level and in 10.1 % of those treated at the lumbar level. The incidence of complications for all levels of surgically treated spine was higher than that observed in the 2001 survey [5].

### Relationship between surgical approach the incidence of complication

Complications occurred in 10.2 % of patients treated by a posterior approach, 14.5 % of those treated by an anterior approach and 27.7 % of those treated by a combined anterior/posterior approach (*p* < 0.05). These findings were similar to those reported in the 2001 survey (Table 10) [5].

**Table 10:** Relationship between surgical approach and the incidence of complications

	Approach		
	Anterior (%)	Posterior (%)	Combined anterior/posterior (%)
2011	14.5	10.2	27.7
2001	13.9	7.9	17.0

### Relationship between surgical approach, the incidence of complication and the level of the spine

Table 11 shows the relationship between surgical approach, incidence of complications and level of the spine. The highest incidence of complication was observed for a combined anterior/posterior approach in the thoracic spine (31.0 %). Complications for the cervical, thoracic and lumbar spine levels were related to the surgical approach (each *p* < 0.05).

**Table 11:** Relationship between surgical approach and the incidence of complications and level of the spine

	Approach		
	Anterior (%)	Posterior (%)	Combined anterior/posterior (%)
Cervical spine	12.9	11.4	23.7
Thoracic spine	22.2	13.8	31.0
Lumbar spine	19.2	9.7	29.4

### Details of complications

Table 12 shows the incidence of complications in all spine surgery in the 1994, 2001 and 2011 surveys. The highest incidence of complication was dural tear (2.1 %), followed by neurological complication (1.4 %), DWI (1.1 %) and EH (0.9 %). The incidence of neurological complications (1.4 %) was higher than in the 1994 survey (0.9 %, *p* < 0.05), but lower than in the 2001 survey (1.7 %, *p* < 0.05) [4, 5]. The incidence of DWI has increased progressively from 0.6 % in the 1994 survey to 0.9 % in the 2001 survey and 1.1 % in the 2011 survey (*p* < 0.05 for 1994 vs. 2011) [4, 5]. The incidence of mental disorders in the 2011 survey increased compared to both earlier surveys (each *p* < 0.05) [4, 5]. The incidence of death increased from 0.08 % in the 2001 survey to 0.2 % in 2011 (*p* < 0.02). The incidence of PE/TE increased from 0.1 % in the 2001 survey to 0.2 % in the 2011 survey (*p* < 0.05) [5]. The incidence of vascular injury decreased from 0.05 % in the 2001 survey to 0.02 % in 2011 (*p* = NS) [5].

**Table 12:** Details of complications

Complication	2011		2001		1994	
	No. of cases	Incidence (%)	No. of cases	Incidence (%)	No. of cases	Incidence (%)
Dural tear	661	2.1	219	1.4	103	0.6
CSF leakage	168	0.5				
Neurological complications	425	1.4	279	1.7	181	0.9
Deep wound infection	343	1.1	153	0.9	124	0.6
Epidural hematoma	288	0.9			–	–
Implant dislodgement	180	0.6	–	–	–	–
Mental disorder	164	0.5	47	0.3	50	0.3
Respiratory disease	164	0.5	65	0.4	27	0.1
Digestive/liver disease	127	0.4	58	0.4	49	0.3
Circulatory disease	99	0.3	–	–	–	–
Wrong level	60	0.2	–	–	–	–
Pulmonary embolism/thromboembolism	59	0.2	18	0.1	–	–
Death	54	0.2	13	0.08	–	–
Cerebral disease	49	0.2	–	–	–	–
Implant failure	9	0.03	84	0.5	–	–
Vascular injury	7	0.02	8	0.05	–	–
Dislodgement of grafted bone	–	–	42	0.3	34	0.2
Others	643	2.0	315	2.0	–	–

In survey, 231 overlapping cases

*CSF* cerebrospinal fluid

### Details of the neurological complications

Table 13 shows details of the neurological complications. Spinal cord damage occurred in 85 cases (0.3 %), nerve root damage in 297 cases (0.9 %) and cauda equina damage in 50 cases (0.2 %). Seven cases showed damage at two sites. The incidence of damage to the nerve root, spinal cord and cauda equina were all higher in the 2011 survey compared to the 1994 survey, but only the former reached significance (*p* < 0.05) [4].

**Table 13:** Details of the neurological complications

	No. of cases incidence (%)	
	2011	1994
Spinal cord damage	85 (0.3)	48 (0.2)
Nerve root damage	297 (0.9)	103 (0.5)
Cauda equina damage	50 (0.2)	30 (0.2)

Seven cases showed damage at two sites in Survey 2011

### Incidence of complications according to the experience of the surgeon

Table 14 shows the incidence of complications according to the experience of the surgeon. Neurological complications occurred most frequently (2.4 %) in patients treated by surgeons with less than 5 years of experience. In the 2001 survey, the incidence of dural tear was clearly lower for patients operated by surgeons with longer experience in spine surgery [5]. However, in the present survey, dural tear occurred most often in patients treated by surgeons with 10–19 years of experience (incidence of 2.4 %), and was actually lower for surgeons with less experience (2.0 %). DWI occurred most frequently in patients treated by surgeons with 20 or more years of experience (incidence of 1.2 %) and was lowest in patients treated by surgeons with 5 or less years of experience (0.4 %). The incidence of representative complications (neurological complications, dural tear and DWI) was not significantly different between surgeons with different lengths of professional experience.

**Table 14:** The incidence of representative complications according to the experience of the surgeon

Surgeon’s experience (years)	Neurological complication		Dural tear		Deep wound infection	
	Incidence (%)	No. of cases	Incidence (%)	No. of cases	Incidence (%)	No. of cases
<5	2.4	6	2.0	5	0.4	1
5–9	1.1	29	2.0	54	1.1	31
10–19	1.4	180	2.4	313	1.0	133
>20	1.4	209	1.9	289	1.2	177

### Instrumentation surgery

The incidence of complications for cases treated with instrumentation surgery (15.6 %, 1,480/9,487) was almost double that of cases treated by non-instrumentation surgery (8.2 %, 1,789/21,893; *p* < 0.05). This result was similar to the two earlier surveys (15.5 vs. 5.9 % in 1994 and 12.1 vs. 6.8 % in 2001; Table 15) [4, 5]. The incidence of complications with both instrumentation surgery and non-instrumentation surgery was higher in the 2011 survey compared to the two earlier surveys. For patients undergoing instrumentation surgery, this increase occurred regardless of the surgeon’s experience (Table 16). The incidence of complications with instrumentation surgery and with all surgery was highest in patients treated by surgeons with 10–19 years of experience (17.0 and 11.8 %, respectively).

**Table 15:** Incidence of complications with instrumentation surgery and non-instrumentation surgery

	Incidence of complications	
	Instrumentation surgery	Non-instrumentation surgery
2011	15.6 % (1,480/9,487)	8.2 % (1,786/21,893)
2001	12.1 %	6.8 %
1994	15.5 %	5.9 %

**Table 16:** Number of years of experience of the surgeon and incidence of complications

Experience (years)	2011		Experience (years)	2001	
	Incidence of complication			Incidence of complication	
	All surgery	Instrumentation surgery		All surgery (%)	Instrumentation surgery (%)
< 5	10.3 % (26/251)	15.0 % (6/40)	1–6	7.9	12.3
5–9	8.7 % (239/2,754)	12.5 % (104/831)	7–9	8.0	12.3
10–19	11.8 % (1,561/13,212)	17.0 % (682/4,005)	10–14	9.1	13.2
> 20	9.5 % (1,440/15,140)	14.9 % (688/4,606)	>15	8.3	11.0

### Spinal cord damage according to diagnosis and the level of the spine

Spinal cord damage was reported in 85 patients. This was due to a tumor in 21 cases, OPLL in 20 cases and cervical spondylotic myelopathy in 13 cases. Similar to the 1994 survey, an intramedullary tumor was the cause of many of the cases (9/21) with spinal cord damage due to a tumor [4]. Cervical surgery was indicated in 44 cases with spinal cord damage and thoracic surgery in 23 cases.

### Deep wound infection

DWI occurred in 343 (1.1 %) of the 31,380 surveyed cases and was more frequent in those with complicated and invasive surgical procedures. DWI was threefold higher in cases treated with instrumentation surgery (2.0 %, 189/9,487 cases) compared to those treated with non-instrumentation surgery (0.7 %, 154/21,893 cases; *p* < 0.05). In the 1999 survey, DWI was fivefold higher in cases treated with instrumentation surgery (1.0 %, 53/5,210 cases) compared to those treated with non-instrumentation surgery (0.2 %, 27/14,061 cases [4]. The distribution of DWI was: DM (25.1 %), dialysis (2.6 %), corticosteroid use (5.5 %), DMARD therapy involving biological therapy (5.3 %) and Parkinson’s disease (1.8 %). The incidence of DWI was 2.2 % (86/3,792) in cases with DM, 2.1 % (9/437) in cases with dialysis, 2.7 % (19/701) in cases with corticosteroid use, 3.5 % (18/512) in cases with DMARD therapy involving biological therapy and 3.0 % (6/198) in cases with Parkinson’s disease. DWI was significantly associated with each of these conditions (each *p* < 0.05).

### Relationship between operation time and deep wound infection, epidural hematoma, pulmonary embolism and/or thromboembolism and death

After exclusion of 145 cases with missing information on the operation time, a total of 337 cases with DWI, 288 cases with EH, 59 cases with PE/TE and 54 cases who died were evaluated (Table 17). DWI, EH and PE/TE showed significant correlations with the length of operation time (each *p* < 0.05; Spearman’s correlation coefficient).

**Table 17:** Relationship between operation time and deep wound infection, epidural hematoma, pulmonary embolism and/or thromboembolism and death

Operation time (h)	Deep wound infection	Epidural hematoma	Pulmonary embolism and/or thromboembolism	Death
	No. of cases incidence (%)	No. of cases incidence (%)	No. of cases incidence (%)	No. of cases incidence (%)
< 3	175 (0.8)	172 (0.8)	17 (0.1)	24 (0.1)
3–6	135 (1.9)	99 (1.4)	34 (0.5)	25 (0.3)
> 6	27 (2.2)	17 (1.4)	7 (0.6)	5 (0.4)

### Relationship between intraoperative blood loss and deep wound infection, epidural hematoma, pulmonary embolism and/or thromboembolism and death

After exclusion of 192 cases that lacked information on intraoperative blood loss, a total of 343 cases with DWI, 275 cases with EH, 59 cases with PE/TE and 54 cases who died were subjected to analysis (Table 18). DWI, EH, PE/TE and death all showed a significant correlation with volume of intraoperative blood loss (each *p* < 0.05; Spearman’s correlation coefficient).

**Table 18:** Relationship between intraoperative blood loss and deep wound infection, epidural hematoma, pulmonary embolism and/or thromboembolism and death

Intraoperative blood loss	No. of cases incidence (%)			
	Deep wound infection	Epidural hematoma	Pulmonary embolism and/or thromboembolism	Death
<200 ml	162 (0.7)	154 (0.7)	19 (0.1)	21 (0.1)
200–500	101 (1.9)	68 (1.2)	22 (0.4)	18 (0.3)
500–1000	31 (1.9)	23 (1.1)	8 (0.4)	9 (0.4)
1000–2000	21 (1.5)	18 (2.2)	7 (0.8)	4 (0.5)
>2000	12 (3.1)	12 (3.1)	2 (0.5)	2 (0.5)

### Diagnosis and the cause of death

The 54 patients who died were evaluated for the diagnosis and the cause of death (Tables 19, 20). There were 30 males and 24 females and the mean age was 69.9 years (range 38–91 years). The highest incidence of death was associated with dialysis (4.0 %, 4/101) followed by metastatic spine tumor (3.9 %, 15/389). With regard to the cause of death, tumor-associated death was the most common, followed by inflammation of the lungs.

**Table 19:** Diagnosis of 54 cases with death

	No. of cases
Metastatic spine tumor	15
Inflammation	
Pyogenic infection	6
Rheumatoid arthritis	4 (O-C fusion 3, O-T fusion 1)
Tuberculous infection	1
Dialysis	4
Trauma	
Cervical spine	6
Lumbar spine	1
Osteoporotic vertebral collapse	4
Degenerative disease	
Stenosis	
Lumbar spine	3
Cervical spine	2
Degenerative spondylolisthesis (lumbar spine)	1
Ossification of ligaments	
OPLL (cervical spine)	1
OLF (lumbar spine)	1
Primary malignant spine tumor	2
Intramedullary tumor	1
Epidural hematoma	1
Spinal deformity Scoliosis	1

*OPLL* ossification of posterior longitudinal ligament, *OLF* ossification of ligamentum flavum, *O-C fusion* occipito-cervical fusion, *O-T fusion* occipito-thoracic fusion

**Table 20:** The cause of death

The cause of death	No. of cases
Tumor-associated death	16
Inflammation of the lungs	5
Sepsis	4
DIC	3
Pulmonary embolism	3
Liver failure	2
Cerebral infarction	1
Myocardial infarction	1
Respiratory problem	1
Unknown cause	18

*DIC* disseminated intravascular coagulation

### Relationship between the incidence of representative complications and the surgical technology used

Table 21 shows the relationship between the incidence of representative complications and the surgical technology used. The incidence of dural tear was low (1.5 %) for microscopic surgery, while the incidence of EH was high (1.1 %) for conventional surgery. The incidence of DWI (0.05 %) and the overall incidence of complications were very low for endoscopic surgery. Surgeons with longer experience in spine surgery used endoscopic surgery more often.

**Table 21:** Relationship between the incidence of representative complications and the surgical technology used

Representative complications	No. of cases incidence (%)			
	Conventional (20,697 cases)	Endoscopic (4,110 cases)	Microscopic (6,523 cases)	Percutaneous (501 cases)
Spinal cord damage (85 cases: overlapping 3 cases)	66 (0.3)	0 (0.0)	22 (0.3)	0 (0.0)
Nerve root damage (297 cases: overlapping 8 cases)	244 (1.2)	22 (0.5)	38 (0.6)	1 (0.2)
Cauda equina damage (50 cases: overlapping 2 cases)	38 (0.2)	4 (0.1)	10 (0.2)	0 (0.0)
Dural tear (685 cases: overlapping 24 cases)	486 (2.3)	100 (2.4)	98 (1.5)	1 (0.2)
Epidural hematoma (288 cases: overlapping 6 cases)	227 (1.1)	23 (0.6)	44 (0.7)	0 (0.0)
Deep wound infection (343 cases: overlapping 6 cases)	299 (1.4)	2 (0.1)	42 (0.6)	6 (1.2)

### Relationship between the incidence of representative complications and the surgical technology used for patients with lumbar disc herniation at the L4/5 level

Table 22 shows the association between the incidence of representative complications and the surgical technology used for 3,415 patients with lumbar disc herniation at the L4/5 level. Of these, 1,352 were treated by conventional surgery, 1,002 by endoscopic surgery, 1,044 by microscopic surgery and 17 by percutaneous surgery. Excluding the latter 17 patients, the complications of nerve root damage, EH and DWI were significantly correlated with surgical technology (each *p* < 0.05), whereas the complications of cauda equina damage and dural tear showed no significant correlation with surgical technology.

**Table 22:** Relationship between the incidence of representative complication and the surgical technology used for patients with lumbar disc herniation at L4/5 level

Representative complications	No. of cases incidence (%)			
	Conventional (1,352 cases)	Endoscopic (1,002 cases)	Microscopic (1,044 cases)	Percutaneus (17 cases)
Nerve root damage	12 (0.9)	2 (0.2)	3 (0.3)	0 (0.0)
Cauda equina damage	3 (0.2)	2 (0.2)	2 (0.2)	0 (0.0)
Dural tear	37 (2.7)	23 (2.3)	25 (2.4)	0 (0.0)
Epidural hematoma	12 (0.9)	1 (0.1)	5 (0.5)	0 (0.0)
Deep wound infection	9 (0.7)	0 (0.0)	1 (0.1)	0 (0.0)

### Relationship between the incidence of intraoperative and postoperative complications and BMI

A total of 24,427 patients with information on BMI were available for data analysis, of which 2,551 experienced intraoperative and postoperative complications. The mean BMI of patients with intraoperative and postoperative complications was 23.7 ± 4.1, compared to 23.7 ± 3.8 for those without complications (*p* = NS). The mean BMI of patients with dural tear and PE/TE was higher than that of patients without intraoperative or postoperative complications, and it was lower with cauda equina damage, respiratory disease and death (each *p* < 0.05).

### Mean age of patients with intraoperative and postoperative complications

The mean age of patients with nerve root damage, dural tear, implant failure, cauda equina damage, EH, DWI, mental disorder, PE/TE, circulatory disease, cerebral disease, respiratory disease and death was higher when compared to patients without intraoperative or postoperative complications (each *p* < 0.05), and was lower with implant failure (*p* < 0.05).

### Factors associated with dural tear, DWI, neurological complications and EH

Logistic regression analyses were performed for exploring factors that are associated with intraoperative and postoperative complications. We focused on the complications that occurred in more than 200 cases (dural tear, WDI, neurological complications and EH). Table 23 showed factors found to be associated with these complications. There were no factors associated with EH.

**Table 23:** Factors associated with dural tear, DWI and neurological complications

Target variables	Explanatory variables	Odds ratio	95 % confidence interval	*p*
Dural tear	Lumbar spine	2.3	1.9–2.8	<0.0001
	Posterior approach	1.9	1.3–2.7	<0.01
DWI	Instrument	2.4	1.8–3.1	<0.0001
	DM	2.3	1.8–3.0	<0.0001
	DMARD therapy involving biologic therapy	2.9	1.5–5.5	<0.0001
Neurological complications	Instrument	1.8	1.5–2.2	<0.0001

*DWI* deep wound infection, *DM* diabetes mellitus, *DMARD* disease-modifying antirheumatic drug

## Discussion

Japan has a declining birthrate and an aging society. At the end of 2010, 23.1 % of the Japanese population was aged > 65 years and 11.4 % was aged > 75 years. The proportion aged > 65 years is projected to increase to 30.5 % by 2025 [2]. In 2010, Japan was the only super aging society amongst advanced nations. Therefore, clinical solutions to intraoperative and postoperative complications from spinal cord and spine surgery in aged patients are urgently required. The most frequent patient age in this 2011 nationwide survey of spine surgery was 70–79 years. A total of 19,802 patients were aged 60 years or older, corresponding to 63.1 % of the overall cohort and higher than the value of 49 % reported in 2001 (*p* < 0.05). In 2001, just 3.8 % of all patients were aged 80 years or older, whereas in 2011 this had increased to 10.0 % (*p* < 0.05) [5]. With regard to diagnosis, the incidence of osteoporotic vertebral collapse and stenosis was higher than in the 2001 survey (*p* < 0.05), reflecting the increased number of elderly patients undergoing spine surgery [5]. The 3,269 patients with complications in the 2011 survey had a mean age of 64.4 ± 17.0 years. Significant differences were apparent between patients with and without complications, especially for mental disorders, death and circulatory disease. Dekutoski et al. [9] reported that complication rates for spine surgery were higher in older patients and in patients with multiple comorbidities, diabetes, obesity or hypertension.

### Surgical approach

Compared to the 1994 and 2001 surveys, the frequency of anterior surgery was lower in 2011, whereas that of posterior surgery increased [4, 5]. Posterior rather than anterior decompression surgery was increasingly used for CSM or CSR and for CDH. In the 2001 survey, complications were more frequent in cases treated by an anterior approach compared to cases treated by a posterior approach, possibly explaining the subsequent decrease in use of this practice [5]. In general, the posterior approach is safer, easier to perform and less invasive than the anterior approach [10]. It is likely that a posterior approach is preferred by many surgeons who treat increased numbers of elderly patients.

In the current survey, the incidence of complications was related to the surgical approach used for the cervical, thoracic and lumbar spine. Surgeons should be especially aware of complications that can arise when using an anterior approach for thoracic spine. The Scoliosis Research Society reported similar complication rates for anterior versus posterior approaches in the treatment of adolescent idiopathic scoliosis [7]. Combined anterior and posterior instrumentation and fusion leads to a significantly higher incidence of neurologic complications than anterior or posterior instrumentation and fusion alone. The incidence of infection with the posterior approach is higher than with the anterior approach [11]. Pull ter Gunne [12] reported that isolated anterior surgical approaches were associated with a 1.7 % risk of surgical-site infection (SSI), whereas any surgery that included a posterior spinal approach was associated with a minimum risk of infection of 4.4 %. Care should therefore be taken to prevent infection when using a posterior approach.

### Surgical technology

Endoscopic and microscopic surgery were indicated in 2.6 and 11.1 % of cases in the 2001 survey, respectively, but increased to 12.9 and 20.5 % in the 2011 survey (each *p* < 0.05) [5]. The overall incidence of complications was very low for endoscopic surgery (Table 21). However, because endoscopic and conventional surgeries are used for different pathologies, it is difficult to correlate the incidence of complications with the surgical technology used. In addition, surgeons with a longer experience in spine surgery tended to use endoscopic surgery more often. We therefore correlated the incidence of representative complications with the surgical technology in patients with lumbar disc herniation at L4/5. The incidence of complications from nerve root damage, EH and DWI was very low for both endoscopic and microscopic surgery. Gotfryd and Avanzi. [13] reported that conventional, microscopic and endoscopic posterior discectomy surgical techniques were all effective for the treatment of single level lumbar disc herniations in patients without degenerative vertebral deformities. Endoscopic and microscopic surgery were superior to conventional surgery with respect to the volume of blood loss, systemic repercussions and duration of hospital stay [14]. The incidence of neurological complications was significantly lower in the 2011 survey compared to the 2001 survey, whereas the incidence of mental disorders, death and PE/TE increased significantly [5].

The incidence of representative complications (neurological complications, dural tear and DWI) did not vary significantly according to the experience of the surgeon.

### Spinal instrumentation

In the current survey, the incidence of complications in patients treated with instrumentation surgery was higher than in those treated with non-instrumentation surgery (*p* < 0.05) and was significantly higher than in the 2001 survey (*p* < 0.05) [5]. This can be explained by the fact that patients indicated for instrument surgery were older. Carreon et al. [15] have previously reported that the complication rate increases with older age, increased blood loss, longer operation time and the number of levels of the arthrodesis. The most common major complication in older patients was wound infection, with a prevalence of 10 % for posterior lumbar decompression and for arthrodesis. These authors suggested that attention should be paid to the control of blood loss, and to limiting the operative time. On the other hand, Cassinelli et al. [16] argued that age should not be used as a criterion to avoid decompression and fusion with or without instrumentation for the treatment of lumbar stenosis associated with instability. Elderly patients can undergo these procedures safely with a low risk of developing major perioperative complications.

### Deep wound infection

It is important to be aware of the risk factors for infection, so that preventive measures can be taken and the surgical treatment optimized [17]. Pull ter Gunne and Cohen [12] reported that blood loss of >1 l, previous SSI and DM were independent risk factors for SSI, while obesity was an independent risk factor for superficial SSI, and DM, obesity, previous SSI and longer surgeries (>5 h) were independent risk factors for deep SSI. In the current survey, we reviewed the preoperative factors of DM, dialysis, corticosteroid use, DMARD therapy involving biologic therapy, Parkinson’s disease, age and BMI. The intraoperative factors reviewed were operation time and intraoperative blood loss. The incidence of DWI was significantly associated with the preoperative factors of DM, dialysis, corticosteroid use, DMARD therapy involving biologic therapy, Parkinson’s disease and age (each *p* < 0.05), as well as with the intraoperative factors of operation time and blood loss (each *p* < 0.05). Factors associated with DWI were instrumentation surgery, DM and DMARD therapy involving biological therapy, as results of multiple logistic regression analyses.

### Epidural hematoma

It is important to be aware of the risk factors for EH so that preventive measures can be taken and surgical treatment optimized. Postoperative spinal EH are very rare, with a reported incidence ranging from 0.1 to 1 % [18–21]. Patients who undergo multilevel operative procedures, are > 60 years old and/or have preoperative coagulopathy have a significantly higher risk [18–20]. In the current survey, we reviewed the preoperative factors of patient age and BMI, while the intraoperative factors reviewed were operation time and intraoperative blood loss. EH was significantly associated with age as a preoperative factor (*p* < 0.05) and with operation time and blood loss as intraoperative factors (each *p* < 0.05). Cabana et al. [22] and Amiri et al. [23] previously reported on the association between surgical time and neurological outcome. Evacuation of EH within 4 h resulted in better neurological recovery than surgery after 4 h. Seichi et al. [24] reported on neurological complications of cervical laminoplasty for patients with OPLL and found that the incidence of EH was 0.5 % (3/581 patients). The three cases with EH were diagnosed on the day of surgery, demonstrating that careful attention should be paid to the condition of patients at that time.

### Pulmonary embolism/thromboembolism

It is also important to know the risk factors for PE/TE so that preventive measures can be taken and surgical treatment optimized. The incidence of PE/TE in the current survey was 0.2 % (59/31,380) compared to 0.1 % (18/16,157) in the 2001 survey [5]. The preoperative factors reviewed in this survey were age and BMI, while the intraoperative factors reviewed were operation time and intraoperative blood loss. PE/TE was significantly associated with age and BMI (each *p* < 0.05), and with operation time and intraoperative blood loss (each *p* < 0.05). However, due to insufficient data, a standardized prophylactic regimen cannot be recommended. Elastic compression alone or combined with pharmacological prophylaxis appears to be effective. Schoenfeld et al. [25] reported that a BMI of 40 or more, age of 80 years or more, operative time exceeding 261 min and American Society of Anesthesiologist classification 3 or greater were each significant independent predictors of deep vein thrombosis. They also reported that a BMI of 40 or more, operative time > 261 min and male gender were associated with the development of PE. Prophylaxis with both measures is strongly recommended for high-risk patients [26]. Dearborn et al. [27] investigated the association between surgical procedures and PE in 318 major spinal reconstructive procedures. They reported seven cases of PE, of which six occurred amongst 97 patients undergoing combined anterior and posterior spinal procedures (6.1 %) and one in a patient undergoing a posterior procedure. The overall clinical PE rate with the combined approach was significantly higher than for patients who underwent the posterior approach only. Preventive measures include pharmacological, mechanical and combinations of these interventions. Mechanical interventions include elastic compression stockings and intermittent pneumatic compression devices. Pharmacological options include low-dose unfractionated heparin or a low molecular weight heparin (LMWH) such as enoxaparin, dalteparin, tinzaparin, certoparin or nadroparin [28]. Because of the possibility of EH, anticoagulation has not gained wide acceptance by spine surgeons [29]. A 2012 survey of British orthopedic spine surgeons revealed that only 31 % routinely used LMWH [30]. Strom et al. [28] reported on the safety and efficacy of prophylactic LMWH (e.g., 40 mg enoxaparin for normal renal function, 30 mg for impaired renal function) starting 24–36 h after multilevel laminectomy or laminectomy and fusion in 367 patients with degenerative disease. None of the patients developed an EH, superficial hematoma or persistent wound drainage. In the current survey, fatal PE was reported in three cases. Surgeons with a better understanding of venous thromboembolism following spine surgery can now weigh the risks and benefits of postoperative anticoagulation measures.

### Limitation

The response was achieved from 209 institutions in 750 institutions (response rate 28 %). Because the response rate was low, the results of this analysis might not reflect correctly the present situation for spine surgery in Japan.

## Conclusions

 In 2011, we carried out the third nationwide survey in Japan on complications from spine and spinal cord surgery and compared the results with previous nationwide surveys conducted in 1994 and 2001 [4, 5]. The most frequent age was in the range of 70–79 years. The diagnosis of osteoporotic vertebral collapse and stenosis has also increased, together with the use of less invasive surgical techniques such as endoscopic and microscopic surgery. The practice of anterior surgery has decreased, while that of posterior surgery has increased. The incidence of neurological complication has significantly decreased, while the incidence of mental disorders, death and PE/TE has increased significantly compared to the 2001 survey. These again reflect the large increase in the elderly patient population. EH, DWI, PE/TE and death were significantly correlated with the volume of intraoperative blood loss and with operation time. Care should therefore be taken to control these factors in order to prevent complications.

The results of this latest nationwide survey should be critically evaluated by members of the JSRS and the Japanese Orthopaedic Association, with the aim of improving therapeutic outcomes for spine and spinal cord surgery. The results should also help to inform patients in the process of obtaining consent for the treatment of spinal diseases.
